# Development of a Risk Score to Predict Detection of Metastasized or Locally Advanced Perihilar Cholangiocarcinoma at Staging Laparoscopy

**DOI:** 10.1245/s10434-016-5531-6

**Published:** 2016-09-01

**Authors:** Robert J. S. Coelen, Anthony T. Ruys, Jimme K. Wiggers, Chung Y. Nio, Joanne Verheij, Dirk J. Gouma, Marc G. H. Besselink, Olivier R. C. Busch, Thomas M. van Gulik

**Affiliations:** 1 Department of Surgery, Academic Medical Center, Amsterdam, The Netherlands; 2 Department of Radiology, Academic Medical Center, Amsterdam, The Netherlands; 3 Department of Pathology, Academic Medical Center, Amsterdam, The Netherlands

## Abstract

**Background:**

Nearly half of patients with perihilar cholangiocarcinoma (PHC) have incurable tumors at laparotomy. Staging laparoscopy (SL) potentially detects metastases or locally advanced disease, thereby avoiding unnecessary laparotomy. However, the diagnostic yield of SL has decreased with improved imaging in recent years.

**Objective:**

The aim of this study was to identify predictors for detecting metastasized or locally advanced PHC at SL and to develop a risk score to select patients who may benefit most from this procedure.

**Methods:**

Data of patients with potentially resectable PHC who underwent SL between 2000 and 2015 in our center were retrospectively analyzed. Multivariable logistic regression analysis was used to identify independent predictors and to develop a preoperative risk score.

**Results:**

Unresectable PHC was detected in 41 of 273 patients undergoing SL (yield 15 %). Overall sensitivity of SL was 30 %, with highest sensitivity for detecting peritoneal metastases (73 %). Preoperative imaging factors that were independently associated with unresectability at SL were tumor size ≥4.5 cm, bilateral portal vein involvement, suspected lymph node metastases, and suspected (extra)hepatic metastases on imaging without the possibility of diagnosis by percutaneous- or endoscopic ultrasound-guided biopsy. The derived preoperative risk score showed good discrimination to predict unresectability (area under the curve 0.77, 95 % confidence interval 0.68–0.86) and identified three subgroups with a predicted low-risk of 7 % (*N* = 203 patients), intermediate-risk of 21 % (*N* = 39), and high-risk of 58 % (*N* = 31).

**Conclusions:**

A selective approach for SL in PHC is recommended since the overall yield is low. The proposed preoperative risk score is useful in selecting patients for SL.

**Electronic supplementary material:**

The online version of this article (doi:10.1245/s10434-016-5531-6) contains supplementary material, which is available to authorized users.

Perihilar cholangiocarcinoma (PHC) is a rare disease with a dismal prognosis for which radical resection remains the only curative treatment.^1^ Unfortunately, nearly half of patients subjected to laparotomy have unresectable tumors, despite extensive preoperative radiological staging.^2^,^3^ Staging laparoscopy (SL) in addition to imaging prior to exploratory laparotomy may detect small liver metastases or peritoneal metastases, thereby avoiding unnecessary laparotomy. However, the true additional diagnostic value of this procedure remains unclear, with varying results reported in the literature.^4^ Currently, the routine use of SL in preoperative staging of PHC is being questioned as the majority of studies from recent years have shown a diagnostic yield below 20 % and a sensitivity to detect unresectable disease lower than 60 %.^5^–^9^

As the routine use of SL in PHC patients does not seem justified, a selective approach to identify patients who may benefit most from this procedure seems warranted.^5^ There is currently no evidence for adequate selection criteria for SL in PHC patients as most studies are hampered by small sample size, resulting in low predictive power. The aim of this study was therefore to identify predictors of unresectable disease at SL and develop a preoperative risk score in a large cohort of PHC patients treated in a single center specializing in the management of PHC.

## Methods

### Study Population and Selection

Consecutive patients with suspected PHC^10^ who were seen at the Academic Medical Center (AMC) in Amsterdam between May 2000 and July 2015 were identified from a prospectively maintained database. Exclusion criteria were SL for gallbladder carcinoma, and distal or intrahepatic cholangiocarcinoma.

All patients and radiological scans were discussed in a multidisciplinary, hepatopancreatobiliary (HPB) team meeting. In the event of suspected distant lymph node or organ metastases, percutaneous- or endoscopic ultrasound (EUS)-guided biopsy was attempted in order to confirm metastatic disease. When tumors were considered potentially resectable, SL was planned early in the preoperative workup in all patients with Bismuth–Corlette (BC) type 3–4 tumors. Laparoscopy for BC type 1–2 tumors was conducted more selectively according to the surgeon’s preference. Laparoscopy was also performed in the event of suspicious metastatic lesions on imaging for which diagnosis by percutaneous- or EUS-guided biopsy was not feasible or when pathological results of biopsies were inconclusive with persistent suspicion of metastatic disease.

### Staging Laparoscopy (SL) and Exploratory Laparotomy

SL was carried out by the HPB surgical fellow or surgeon and included inspection of the liver and gallbladder surface, peritoneal cavity, and hepatoduodenal ligament.^5^,^11^ Lymph node sampling was not performed routinely, but only for enlarged or suspicious lymph nodes. Furthermore, the lesser omentum was not routinely opened, but if done so, the common hepatic artery was inspected along its course, in search of suspicious lymph nodes. Laparoscopic ultrasound (LUS) was only performed in four patients within the study period.

Patients were scheduled for exploratory laparotomy when no metastases or locally advanced tumors were found at SL, when any new imaging did not show disease progression, and if the patient remained fit to undergo surgery. At laparotomy, the abdomen was inspected for any signs of incurable disease, such as peritoneal deposits, liver metastases, lymph node involvement beyond the hepatoduodenal ligament (N2), or locally advanced disease. Tumors were considered locally advanced if they invaded surrounding organs or when excessive vascular or biliary involvement precluded an R0 or R1 resection and only R2 resection was possible. Portal vein reconstructions were performed if necessary and technically feasible. All suspicious lesions and lymph nodes were biopsied and analyzed by frozen-section microscopic examination.

### Definition of Potential Risk Factors

Study variables included clinical variables [jaundice, carbohydrate antigen (CA) 19-9] and radiological parameters that were retrospectively rereviewed on available computed tomography (CT) and magnetic resonance imaging (MRI) scans by an experienced staff radiologist (CYN) who was blinded to the outcome of SL.

Radiological parameters included BC classification, tumor size, vascular involvement, lobar atrophy, suspected lymph node metastases, and other suspicious metastatic lesions (intrahepatic, peritoneal). Suspicious lymph nodes were larger than 10 mm in short-axis diameter, had irregular border, or showed central necrosis and were categorized as perihilar (N1) or beyond the hepatoduodenal ligament (N2).^10^,^12^ Vascular involvement was defined as more than 180 degrees circumferential tumor contact or as clear distortion, narrowing or occlusion of the portal venous system and/or (branches of) hepatic artery.^13^ Lobar atrophy was defined as a small, hypoperfused lobe with crowding of dilated intrahepatic bile ducts. To avoid misinterpreting intrahepatic cholangiocarcinoma as PHC, we adhered to previously published anatomical landmarks.^14^

### Statistical Analysis

The diagnostic accuracy of SL was expressed as the yield and sensitivity to detect incurable disease. The yield and sensitivity were calculated by dividing the total number of avoided laparotomies by the total number of laparoscopies and all patients with unresectable disease, respectively.

Logistic regression analysis was used to identify predictors of unresectable disease at SL. All study variables with a *p* value < 0.100 in univariable analysis were subsequently entered in multivariable analysis. A cut-off for tumor size was used in these analyses and determined at the highest sensitivity and specificity. A standard approach was then used to develop a risk score.^15^ Independent predictors (*p* < 0.05 in multivariable analysis) were selected and regression coefficients of these predictors were transformed into simple points to develop the risk score. The number of points assigned to each predictor equaled its regression coefficient in multivariable analysis divided by the predictor with the smallest absolute number of points, and rounded to the nearest whole number. Predicted risks were then calculated for each patient in the study cohort by applying total point scores to the logistic regression formula. Risk score tertiles were used to categorize patients into low-risk, intermediate-risk, and high-risk groups based on the predicted chance of finding metastases or locally advanced disease at SL.

Predictive accuracy (discrimination) of the risk score was assessed using area under the curve (AUC) analysis with 95 % confidence intervals (CIs). The agreement between predicted and observed unresectability (calibration) was assessed using the Hosmer–Lemeshow goodness-of-fit test, with a significant outcome (*p* < 0.05) indicating a lack of fit. Missing data were rare and were handled with complete case analysis. All analyses were performed using IBM SPSS Statistics version 22.0 (IBM Corporation, Armonk, NY, USA) and R Version 3.1.2 (R Foundation for Statistical Computing, Vienna, Austria).

## Results

### Patient Demographics and Imaging

**Fig. 1:**
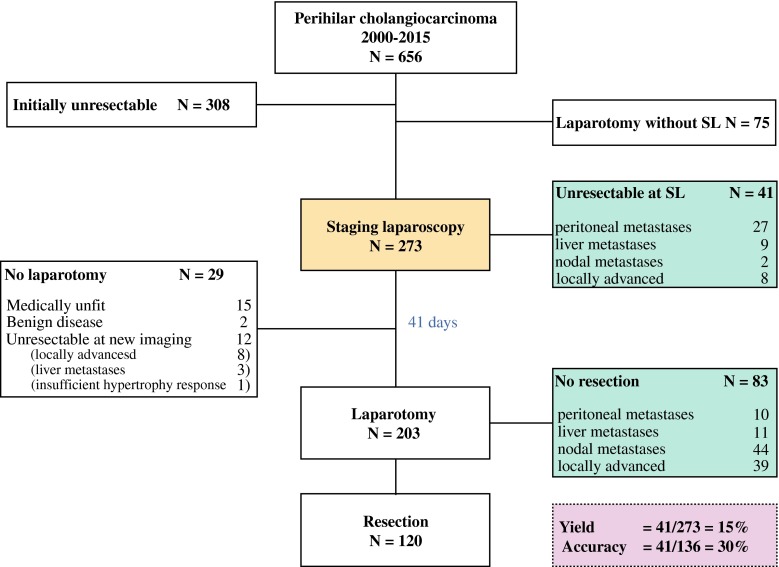
Outcomes of patients with suspected perihilar cholangiocarcinoma undergoing staging laparoscopy and exploratory laparotomy at the Academic Medical Center between 2000 and 2015. *SL* staging laparoscopy

A total of 656 patients with suspected PHC were identified within the study period. After multidisciplinary team discussion, 348 tumors were found to be potentially resectable and 273 patients underwent SL (Fig. 1). Preoperative staging was performed with ultrasound, CT in most patients (97 %), and more selectively with MRI (38 %) and positron emission tomography (PET; 22 %). Patient characteristics are presented in Table 1. Seventy-five patients were directly scheduled for exploratory laparotomy (electronic supplementary Table).

**Table 1:** Baseline characteristics of the study cohort

	Patients (*n* = 273)
Age, years [mean (SD)]	65 (11)
Jaundice at presentation	235 (86.1)
CA19-9, kU/L [median (range)]	172 (1–51,046)
Preoperative staging	
CT	266 (97.4)
MRI	103 (37.7)
US duplex	73 (26.7)
PET	61 (22.3)
Tumor size, cm [mean (SD)]	2.8 (1.3)
≥4.5	25 (9.2)
Bismuth–Corlette type	
I	12 (4.4)
II	20 (7.3)
IIIa	112 (41.0)
IIIb	60 (22.0)
IV	64 (23.4)
Left or right duct	5 (1.8)

Data are expressed as number of patients (%), unless stated otherwise

*SD* standard deviation, *CA19-9* carbohydrate antigen 19-9, *CT* computed tomography, *MRI* magnetic resonance imaging, *US* ultrasound, *PET* positron emission tomography

### Surgical Findings

Inoperable tumors were found during SL in 41 patients, resulting in a yield of 15 % (95 % CI 11–19). Lesions that were mainly detected were peritoneal and liver metastases. Twenty-nine patients who were considered resectable at SL did not undergo laparotomy (Fig. 1). After a median of 41 days (range 3–156), exploratory laparotomy was performed in 203 patients and showed unresectable disease in 83 patients (41 %), mainly because of lymph node metastases and locally advanced tumors that were not apparent on imaging or during SL (Fig. 1). SL correctly identified 41 of 136 patients with unresectable PHC, resulting in an overall sensitivity of 30 % (95 % CI 22–38). Highest sensitivity was found for peritoneal metastases (27/37, 73 %), while sensitivity for detecting liver metastases was 39 % (9/23). Locally advanced tumors (8/55, 15 %) or lymph node metastases (2/46, 4 %) were hardly detected by SL. Of all 44 positive N2 lymph nodes that were found at laparotomy, 29 were located alongside the common hepatic artery.

Complications after SL occurred in eight patients (3 %) and were all minor (Clavien–Dindo grade I–II) and included urinary retention (*N* = 3), pneumonia (*N* = 1), pain requiring prolonged hospital stay (*N* = 1), PTC drain dislocation (*N* = 1), and fever requiring antibiotics (*N* = 2). Complications after exploratory laparotomy occurred in 27 patients (33 %) with unresectable disease, and included 10 major complications (including one death). Median hospital stay for SL was 3 days (range 1–9), including the day of admission.

### Preoperative Predictors of Unresectable Tumors at SL

Univariable and multivariable analysis of predictors for detecting metastasized or locally advanced PHC at SL are shown in Table 2. Independent predictors (*p* < 0.05 in multivariable analysis) that were identified were tumor size (≥4.5 cm), portal vein involvement (bilateral or main stem), suspected metastases in N2 lymph nodes, and suspected (extra)hepatic metastases. No association was found for proximal extent of bile duct involvement (BC classification).

**Table 2:** Univariable and multivariable analysis of risk factors for detecting unresectable perihilar cholangiocarcinoma at staging laparoscopy

Variable	Patients	Univariable analysis		Multivariable analysis	
	[*n* (%)]	OR (95 % CI)	*p* value	OR (95 % CI)	*p* value
Jaundice at presentation	235 (86.1)	1.6 (0.5–4.7)	0.407	–	
CA19-9	–	1.0 (1.0–1.0)	0.764	–	
Bismuth–Corlette type				–	
I	12 (4.4)	Reference			
II	20 (7.3)	1.7 (0.3–10.3)	0.583		
IIIa	112 (41.0)	0.7 (0.1–3.3)	0.612		
IIIb	60 (22.0)	0.6 (0.1–3.2)	0.507		
IV	64 (23.4)	1.3 (0.3–6.5)	0.771		
Left or right duct	5 (1.8)	3.3 (0.3–34.8)	0.315		
Tumor size, cm					
<4.5	248 (90.8)	Reference			
≥4.5	25 (9.2)	3.8 (1.6–9.3)	0.004	4.1 (1.4–11.8)	0.008
Portal vein involvement					
None	151 (55.3)	Reference			
Unilateral	98 (35.9)	1.1 (0.5–2.4)	0.754	1.1 (0.5–2.7)	0.830
Bilateral or main stem	24 (8.8)	5.3 (2.0–13.6)	0.001	3.9 (1.3–12.2)	0.021
Hepatic artery involvement					
None	175 (64.1)	Reference			
Unilateral	89 (32.6)	1.5 (0.7–3.1)	0.279	1.3 (0.5–3.0)	0.564
Bilateral or main stem	9 (3.3)	9.2 (2.3–36.9)	0.002	4.2 (0.7–23.4)	0.105
Suspected lymph node metastases					
None	181 (66.3)	Reference			
N1	73 (26.7)	1.2 (0.5–2.7)	0.645	0.6 (0.2–1.7)	0.345
N2	19 (7.0)	8.5 (3.1–23.2)	<0.001	4.9 (1.4–16.6)	0.012
Suspected (extra)hepatic metastases	22 (8.1)	9.2 (3.7–23.2)	<0.001	9.3 (2.9–30.4)	<0.001
Lobar atrophy	63 (23.1)	0.8 (0.3–1.8)	0.558	–	

Suspected metastases were suspicious metastatic lesions on imaging for which diagnosis by percutaneous biopsy was not feasible or when pathological results of biopsies were inconclusive with ongoing suspicion of metastases. N2 lymph nodes were located beyond the hepatoduodenal ligament

*OR* odds ratio, *CI* confidence interval, *CA19-9* carbohydrate antigen 19-9

There was no difference in the yield of SL in patients who underwent MRI (16/103, 15.5 %) compared with those who did not undergo MRI (25/170, 14.8 %).

### Preoperative Risk Score

The derived preoperative risk score to predict detection of metastasized or locally advanced PHC at SL is presented in Table 3. The sum of the risk score ranges between 0 and 5 points, and predicted risks for each point score are presented in Table 4. The predicted risk was 7.2 % in the low-risk tertile (0 points, *N* = 203 patients), 21.3 % in the intermediate-risk tertile (1 point, *N* = 39), and 58.0 % (range 48.5–91.9 %) in the high-risk tertile (≥2 points, *N* = 31).

**Table 3:** Preoperative risk score to predict unresectable perihilar cholangiocarcinoma at staging laparoscopy

Variable	Classes	Points
Tumor size, cm	<4.5	0
	≥4.5	1
Portal vein involvement	None or unilateral	0
	Bilateral or main stem	1
Suspected lymph node metastases^a^	None or N1 lymph nodes	0
	N2 lymph nodes	1
Suspected (extra)hepatic metastases^a^	No	0
	Yes	2

^a^Ongoing suspicion on lymph node or (extra)hepatic metastases after previous inconclusive/negative biopsy

**Table 4:** Predicted and observed risks according to the risk score points

Group	Point total	*N*	Unresectability at SL	
			Predicted (%)	Observed (%)
Low-risk	0	203	7.2	6.4
Intermediate-risk	1	39	21.3	28.2
High-risk	2	21	48.5	47.6
	3	9	76.5	66.7
	4	1	91.9	100
	5	0	NA	NA

*N* number of patients in the study for each point score, *NA* not applicable, *SL* staging laparoscopy

Predictive performance of the preoperative risk score was well, with an AUC of 0.77 (95 % CI 0.68–0.86) and excellent calibration was observed (Hosmer–Lemeshow test *p* = 0.995). Predictive accuracy remained good after categorizing patients into low-risk, intermediate-risk, and high-risk groups (AUC 0.77, 95 % CI 0.68–0.86).

## Discussion

This is the largest study reporting on the use of SL in PHC. In 273 consecutive patients, a relatively low yield (15 %) and overall sensitivity (30 %) of SL were found to detect unresectable PHC. Several independent risk factors were identified that accurately predicted detection of metastases or locally advanced tumors. A preoperative risk score was developed that showed good discrimination to predict unresectable PHC at SL.

Few reports have studied the additional role of laparoscopy in preoperative staging of PHC, with varying results.^6^,^9^,^16^–^18^ Remarkably, until now only one study was able to identify any predictors for a positive yield.^18^ In that study, a significantly higher yield was found for tumors classified as T2 and T3, rather than T1, according to the Blumgart staging system. This observation was not confirmed in a recent study including 100 patients, but re-evaluation of scans and T staging had been performed in only 38 patients in that cohort.^9^ Notably, only one of three criteria of the Blumgart system (i.e. PV involvement) was associated with unresectability at SL in our analysis. However, the Blumgart staging system was originally developed for predicting resectability in a complete cohort of patients with PHC, and may therefore be less applicable for the decision to perform SL. Variables that were identified as independent predictors in our study and that were included in the risk score were tumor size of 4.5 cm or more, bilateral PV involvement, suspicious lymph node metastases, and suspicious (extra)hepatic metastases on imaging. Suspected metastases involved lesions in which it was not possible to obtain histology by percutaneous approach or when there was an inconclusive biopsy in spite of persistent suspicion. The extent of bile duct involvement was not associated with unresectability at SL, therefore selecting patients for SL based on the BC classification does not seem justified.

In this study, SL had the highest sensitivity for detecting peritoneal metastases, whereas sensitivity for liver metastases, locally advanced tumors, and lymph node metastases was poor. A higher incidence of peritoneal and liver metastases was noted in large tumors and tumors with bilateral PV involvement compared with those without these criteria. In addition, among patients in whom N2 lymph node metastases were suspected on preoperative imaging, a substantial proportion had peritoneal metastases at SL. The reasons for missed superficial liver metastases in this study are not entirely clear as most lesions at laparotomy were located in the anterior liver segments and therefore could have potentially been detected at SL.

The median interval between SL and laparotomy was 41 days and included required time for adequate biliary drainage and/or hypertrophy to occur after portal vein embolization. Metastases may have developed or may have grown substantially to become visible in that time, although PHC is characterized by a rather indolent growth. Ideally, SL and laparotomy would be performed in a single session in order to limit hospital admissions and surgical procedures. However, diagnosis of malignancy is sometimes difficult on frozen sections and biopsy material may require additional (immunohistochemical) staining. In addition, many centers face logistical issues related to anesthetic and operating room time planning, and prefer to perform SL separately from laparotomy. Furthermore, early detection of inoperability allows the timely start of palliative care.

At laparotomy, N2 lymph node metastases were a common finding precluding resection, of which the majority were located along the course of the common hepatic artery. As others confirm the high incidence of unexpected lymph node metastases at laparotomy,^2^ a more extended laparoscopic staging procedure in high-risk patients is recommended. Instead of only visualizing the liver surface and peritoneal cavity, routine opening of the lesser sac, and biopsy of lymph nodes at the anterior side of the common hepatic artery (lymph node station 8a), is currently added in these patients in our department. The incidence of complications following SL was low in this study and included only minor events. However, 20 % minor and 12 % major complications (including one death) were observed following exploratory laparotomy in patients with unresectable PHC. These findings highlight the need for accurate preoperative staging.

LUS was only performed in four patients in this study period as it was previously found to be of limited value in our center.^11^ LUS may theoretically be useful in detecting non-superficial liver metastases or vascular involvement, but obtaining histological evidence in the latter case might be difficult without exploration of the liver hilum. Unfortunately, limited data on the additional value of LUS are available from other studies in the literature.^4^ In only two of six studies, the diagnostic yield of SL was increased with the use of LUS, mainly due to the identification of locally advanced tumors.^16^,^19^

The eventual yield of SL is influenced by the extent of preoperative imaging. In our cohort, most patients underwent CT, whereas MRI and PET–CT were only performed in selected patients. Limited data are available to assume that the additional use of MRI will increase the yield of imaging in potentially resectable PHC. Previous studies have found no differences in the diagnostic accuracy of CT or MRI for detecting lymph node metastases.^20^–^22^ Nonetheless, studies are hampered by their retrospective design and small sample size. The additional value of PET–CT was previously investigated at our center, but all patients had undergone SL in that study.^23^ It was found that the yield of PET–CT after staging using CT and laparoscopy was disappointing because of the low sensitivity for detecting distant metastases and comparable detection of lymph node metastases. However, in a large, prospective study including patients not undergoing SL, it was shown that PET–CT had higher accuracy over CT/MRI in the detection of lymph node metastases and distant metastases.^24^ Furthermore, in that study it was shown that PET–CT revealed unsuspected metastases and clarified indeterminate lesions. Future studies might compare the use of PET–CT and SL in the preoperative staging of PHC. Lastly, EUS is often performed to evaluate suspicious lymph nodes. Although the results of EUS-guided lymph node biopsy vary in the literature, this less-invasive technique seems a preferred step prior to SL;^25^ however, some caution may be advised as tumor tract seeding has been reported.^26^

The present study has several limitations. The relatively few unresectable tumors that were detected at SL consequently provided a low number of events for statistical analysis, which may have led to statistical uncertainty in the multivariable analysis, as reflected by the relatively wide CIs. Second, we were unable to perform external validation of the proposed risk score because of the limited sample size. Future studies that validate the risk score are therefore desirable. Third, as this study comprises a cohort of 15 years, the quality of preoperative imaging varied during the study period and may have subsequently influenced the assessment of vascular involvement or metastases in the early years. However, scans were rereviewed by a specialized radiologist and we also did not note any changes in the diagnostic yield of SL within the study period.

## Conclusions

The results from this study support a selective approach to SL in patients with potentially resectable PHC. The proposed preoperative risk score can be useful in selecting patients who may benefit most from this additional staging procedure. Patients in the low-risk group can proceed to exploratory laparotomy without SL. The use of SL in the intermediate-risk group is debatable, whereas patients in the high-risk group are likely to be diagnosed with incurable disease at SL, thereby avoiding an unnecessary laparotomy.

## Electronic supplementary material

Below is the link to the electronic supplementary material.

Supplementary material 1 (DOCX 15 kb)
