# Supplementary material for: Development of a Risk Score to Predict Detection of Metastasized or Locally Advanced Perihilar Cholangiocarcinoma at Staging Laparoscopy
Source: Ann Surg Oncol. 2016 Sep 1;23(Suppl 5):904–10. doi: 10.1245/s10434-016-5531-6 (PMC5149561; doi:10.1245/s10434-016-5531-6)
Supplement: Supplementary file 1 — Supplementary material 1 (DOCX 15 kb) [file 10434_2016_5531_MOESM1_ESM.docx]

**Supplemental table.** Baseline and imaging characteristics of patients who underwent exploratory laparotomy without staging laparoscopy between 2000 and 2015. Variables are shown as the number of patients (%), unless stated otherwise.

|  | **Patients (*n*=75)** |
| --- | --- |
| **Age, years, mean (sd)** | 64 (10) |
| **Tumor size, cm, mean (sd)** | 2.0 (1.6) |
| Tumor size ≥ 4.5 cm | 5 (6.6) |
| **Bismuth-Corlette type** |  |
| I/II/left/right duct | 34 (45.3) |
| IIIa/IIIb | 37 (49.3) |
| IV | 4 (6.6) |
| **Laparotomy year** |  |
| 2000-2009 | 22 (29.3) |
| 2010-2012 | 14 (18.6) |
| 2013-2015 | 39 (52.0) |
| **Resectability rate** | 50 (66.7) |
| **Reason unresectable (*n*=25)** |  |
| Liver or peritoneal metastases | 11 (44.0) |
| Lymph node (N2) metastases | 7 (28.0) |
| Locally advanced tumors | 7 (28.0) |
